# Haredi Fundamentalism in the State of Israel: How the status quo between state and religion provides ground for a modern religious counter-collective

**DOI:** 10.1007/s41682-022-00139-8

**Published:** 2022-12-05

**Authors:** Eik Dödtmann

**Affiliations:** Filmuniversity Babelsberg KONRAD WOLF, Potsdam, Germany

**Keywords:** Israel, Jewish, Status quo, Haredi, Haredism, Fundamentalism

## Abstract

Since its establishment in 1948, the State of Israel, which defines itself as Jewish-nation state, has been providing Haredi Jewry, also known as ultra-Orthodoxy, with a vast autonomy in education, enabling the development of a Jewish “Society of Learning Men.”

This goes back to the *Status quo* regulations, which blocks the separation of state and religion in the country. In this framework, Haredi Jewry, which was nearly extinct after the Shoah, has developed into a striving and confident fundamentalist religious Jewish movement. At the same time, it has become the demographically most dynamic Jewish current. The influence of Haredi Jewry in Israel is crucial, for its leadership and its members do consider their isolationist, counter-acculturative, anti-modern moulding as the only authentic and “pure” form of Judaism, and they actively combat liberal Jewish interpretations or denominations.

In this paper, a discussion about the definition of Haredism as fundamentalism will be provided. Furthermore, it will be argued that through the basic requirement of the *Status quo* between State and Jewish (orthodox) religion, the Haredi society’s attempt to organize itself as a cultural and communal autonomy has been highly successful also against the background of the societal restrictions of this effort given the dependant relationship that has developed between the Haredi community and general Israeli society. As illustrations for this case study, the educational autonomy, the Haredi judicial power over Jewish and non-Jewish citizens and the struggle over the Shabbat regulations will be examined.

## Introduction

Haredi Jewry, known under the popular term “Jewish ultra-Orthodoxy,” is the isolationist, counter-acculturative, anti-modern expression of Judaism. Before the start of the discussion, just a short explanation of the two terms, which will be used equally in this article: The word “ultraorthodox” has been known since the middle of the 20th century—initially from usage in the USA (Blutinger 2007). By then, the self-designation of strictly religious Jews has simply been “religious Jews,” “God-fearing Jews” or “Haredim,” i.e., as “those trembling before the words of God” (Book of Isaiah 66:5). Increasingly since the mid-20th century—in distinction to the religious Zionist and to Modern Orthodox groups—the ultraorthodox Jewish communities in Israel and worldwide refer to themselves mainly as “Haredim.” In addition, strictly religious use as self-reference and as distinction from other Jewish identities the terms “righteous Jews” or “pious Jews,” or the Hebrew expression “Bney Torah” (Sons of the Torah) (Heilman and Friedman 1994, p. 198–199).

Today, Haredi Judaism is the fastest-growing Jewish religious streaming in the 21st century. In Israel, it enjoys a special status as a culturally protected minority (Stopler 2016). Due to the central role of the Jewish religion as a toolbox for Zionism and for the nation-building process and due to the deep interconnection of state and religion, Haredi Judaism was able to quickly recover from the Shoah and to flourish in an unprecedented manner. This can be seen demographically, politically, and culturally. Demographically, the development of Haredi Jewry is well-documented. In Israel of the year 2020, 1,175,088 Jews in Israel were officially listed as “ultraorthodox” (Cahaner and Malach 2020, p. 11), i.e., 14% of the Jewish population. The Haredim are the fastest-growing Jewish community (Cahaner et al. 2017, p. 14), as is the case in other industrialized countries with a noteworthy Jewish population. Demographic projections predict that by 2050, Haredi communities will be the majority of Jews in the USA and in the UK (The University of Manchester 2007). The reasons for this development are multifold. In Israel, since the year 2000, Haredi population has been growing by approximately 4% per year—in comparison, the overall Jewish population has grown by only 1.4% annually. Haredi Israeli women have 6.6 children on average (Cahaner and Malach 2020), Haredi youngsters marry at a low age, and they are ideologically and religiously driven by efforts to rebuild traditional Jewish life after the Shoah and to follow the divine biblical command in Genesis 1:28, to “be fruitful and multiply.” The Haredim are also the youngest population in Israel: by the late 2010s, nearly 60% of its members were under the age of 16. Among the general population, this age-group just counted 30%. At constant birth rates the number of ultraorthodox Jews will be between 1.5 to 1.8 million by 2030 and around 4 million by the year 2050 (Bystrov and Soffer 2012, p. 60).

Cooperation between the state and ultraorthodox Jews has clear limits. In education: Haredim do not attend secular schools within the plethora of different school systems (the others being state-secular, state-religious, and state Arab schools), but they send their children to the institutions of their own educational sector. In the army: While military service is in fact obligatory for all male and female Jewish Israelis, the vast majority of Haredi men and no Haredi women at all do *not* perform military service at arms. This is because the environment in the Israeli army is considered incompatible for the strict rules of Haredi society—observance of Shabbat, separation of sexes, the dietry laws of kashrut, etc. In the judicial system: The Haredi community has established a private court system that adjudicates matters according to Jewish religious law, the Halakhah, rather than state civil law.

The rapidly growing Haredi population poses major political, economic and social challenges for the Israeli society. Politically, because of the growing influence in politics and law-making. Culturally, in maintaining an interpretation of Jewish religion that discriminates women, homosexuals, non-orthodox Jews and non-Jews. Materially, because the large number of Haredi families need housing, schools and jobs. Within Jewish-Israeli society, the risk of poverty is highest among the ultraorthodox population. This is due to the fact, that Haredi men and women are less integrated into the labour market and earn much less than their secular or religious Zionist Jewish fellows.

A very small group of Haredi Jews has maintained the traditional anti-Zionism of the ultraorthodox communities of Eastern Europe into the 21st century. Their umbrella organization—the Neturey Karta (Guardians of the City)—relies on active political means such as propaganda, demonstrations, and agitation, and, in some cases, limited violence in the fight against what they see as an illegitimate state. Another form of “active,” but this time pro-Zionist fundamentalism is practiced by the Chabad-Lubavitcher Hasidic group. Chabad follows the messianic ideology that the return of the Jewish Messiah could be hastened and eventual redemption brought about if all Jews practiced the rituals of Judaism. Thus, many Chabad adherents see their main task in transforming secular Jews into Lubavitcher Hasids (or at least into practicing Jews) and encouraging the non-Jewish nations of the world to observe the Seven Noachide commandments (Heilman 2005). However, as mentioned, Neturey Karta and Chabad do not represent all of today’s Haredi Jewey. The majority of the Haredim, though ideologically not identifying with the secular aspects of the state of Israel, have pragmatically become an integral part of Israeli society and politics. In the following, explanations for this development will be provided.

## The status quo and Jewish national identity in Israel

With the emergence of modern nation-states, religion has had to relinquish its historical hegemonic position in society and thus can serve as a mere historical symbol and source of political legitimacy (Gellner 1990, p. 75–79). It is precisely this loss of hegemony and the seemingly inferior status that goes with it that fundamentalist religious movements seek to resist, to try to return religion to its dominant role and to base politics in a modern nation-state on religious rather than secular principles (Fisher 2016, p. 531). To a certain extent, this is the case in Israel, but under different preconditions. While in modernity Christianity had (and still has) a hegemonic position in most European or Latin American countries, Judaism has not been in the situation of being a state’s religion over the last 1000 years or so (Sand 2009).

Today, many countries separate state and religion, e.g. the USA, France, Sweden or Japan. In Israel such a separation does not exist. Here, the Jewish religion and Haredi Judaism enjoy a privileged position which goes back to the *Status quo*. This informal agreement was sealed between the secular Zionist leadership in the New Yishuv (the Jewish settlement in Palestine under British colonial rule from 1917–1948), and the two Jewish denominations in Palestine right before the establishment of the state. Keen to get the international support of the world’s orthodox Jews, the Jewish Agency of Palestine—as leading international broker on behalf of the Zionist movement—promised in a letter from June 19, 1947, to settle heavily disputed questions of religious practise. Four aspects were negotiated and adopted for the further state: A) The personal status of a Jew should be governed by Jewish law (Halakhah), B) the Shabbat should become the legal day of rest, C) public institutions shall offer kosher food, and D) religious Judaism shall be able to operate an autonomous educational system.

As a consequence, personal status matters—marriage, divorce, and Jewishness as such—have been governed by state-appointed Jewish religious courts. Since their establishment, the majority of the judges in these courts have been coming from Haredi or religious Zionist background. Thus, both leading orthodox religious Judaic streams, the Haredim and the religious Zionists, have been given religious hegemony and certain legislative power within the state. It is also the first time in modern history, that decision-makers of the ultra-Orthodoxy were given legal power over the concerns of a broad Jewish and non-Jewish society. As a consequence, in the Jewish nation-state all Jewish citizens—secular and religious alike—are confronted with the Haredi rabbis’ interpretation of personal status matters. By that, they are hindered from enjoying the full democratic right of getting married without any limitation due to race, nationality or religion, and are prevented from easily founding a family with whom they desire.

The lack of separation of state and religion has further consequences as it led to the intermingling of confessional belonging and citizenship in the state of Israel. In its legislation^1^, the state is defined as the property of the “Jewish people,” a term that is not subject to a clear definition. In the context of the Jewish national ideology, Zionism, the term “Jewish nation” was moulded and ever since has been adequately used. In contrast to the concept of a “Jewish nation,” an “Israeli nation” does not exist in Israeli law. Non-Jewish citizens in Israel, i.e., Palestinians, are attributed to “non-Jewish nations” allocated outside of Israel and only tolerated as “national minorities.”

The concept of the Jewish nation departs from the traditional meaning of the Jewish people as an ethno-religious community, towards that of an ethno-religious nation (Sand 2009). As Jewish nation-state, Israel draws its legitimacy out of the religious foundations of Judaism, the Torah, and from the divine promise given to the mythological biblical figures of Abraham, Moses (Gen 15:18–21), Isaac (Gen 26:3), and Jacob (Gen 28:13)^2^, from the Jewish entities existing in the ancient biblical land (Israel Ministry of Foreign Affairs 2003), and from religious sentiments of Jews in the Diaspora for a return to Jerusalem, the ultimate spiritual center of Judaism. Belonging to the “Jewish nation” means a “birth right” to live and get citizenship of the state of Israel.^3^ Still, joining the “Jewish nation” can be done only by becoming a member of the “Jewish people,” i.e., the religious concept of the Jewish collective. Becoming a member of the “Jewish people” can only take place in the frame of Jewish religious law, which in Israel is dominated by the two orthodox Judaic currents—Haredi Judaism and religious Zionism. While the Jewishness of a person is accepted by both currents, when he/she was born to a halakhikally approved Jewish mother, the question of conversion to Judaism is heavily contested. According to their claim to sole custody of Jewish identity, Haredi rabbis in the state religious courts fight the attempts of progressive Jewish denominations to widen the concepts of Jewishness.

As all four conditions of the aforementioned *Status quo* letter from 1947 eventually became part of the Israeli legal system, Judaism has *de facto* become state religion in Israel—without calling it by name. For the definition of Judaism in Israel fluctuates between “ethnicity” and “religion” the idea of an ethno-religious Jewish nation-state is in contradiction with the idea of democracy and with liberal rights to individuals, as claimed in the Israeli legislation. Hence, political sociologists discuss whether the semi-democratic constellation in the state of Israel fulfills the case of an *ethnic democracy* (Smooha 1997) or rather that of an *ethnocracy* (Yftachel 2006).

In the beginning of the 21st century, the Haredi mainstream does not openly support this political reality, but somewhat does profit from it and is eager to preserve it. By 2020, nearly every third Jewish settler in the Occupied Palestinian Territories of the West Bank and of East Jerusalem, around 130,000 people, was a member of the Haredi community. Though, in contrast to militant religious Zionists, Haredim restrain in their support for the settlement project and they do not exert violence against the Palestinian population. The main reason for the growing number of Haredi Jewish settlers is the need of affordable housing for the fast growing population. Still, the political stance on the topic of the Occupied Territories have been shifting among Haredim over the last years. While in the 1990s, Haredi leaders such as Elazar Menachem Schach and Ovadia Yosef were supportive of a two-state solution under the slogan “Land for Peace” and of an internationally aspired Two-State solution, it is suggested by research, that due to the demographic situation, the Haredi political parties are going to refuse this prospect in the future (Cahaner 2016).

## Haredi jewry—A clarification of terms

There is disagreement in research as which currents of Judaism in the late 20th and early 21st centuries should be designated as “fundamentalist.” While some scholars totally reject the idea of “Jewish fundamentalism,” others have examined Jewish religious currents meticulously and unconditionally, coming to widely appreciated results. By the early 1990s, a certain consensus on the question of “Jewish fundamentalism” was reached. As part of the international *Fundamentalism Project*, two modern forms of Jewish religion were studied and categorized as “fundamentalist”: 1) the militant religious Zionism which emerged after Israel’s conquest of East-Jerusalem and the West Bank in 1967, and 2) Haredi Judaism, i.e., the Jewish ultra-Orthodoxy The first one, militant religious Zionism, is the idea that the so-called “Greater Land of Israel”—the territory of historical Palestine—is holy to the Jewish people to the same degree as the Torah, the Jewish sacred text. Therefore, supporters of militant religious Zionism strive for the possession and “judaization” of the “Land of Israel”—also outside the internationally acknowledged borders of Israel as of 1967—by using violent means against the Palestinian natives and in some cooperation with the institutions of the state of Israel (Aran 1994).

The second fundamentalist religious current in recent Judaism, Haredism, will be discussed in the following. The question of Haredi Jewish identity has long preoccupied both the Haredi public and academic research, especially in light of the demographic developments in Israel. Unlike the question “Who is a Jew?” which has a fixed definition under Jewish religious law—namely: the child of a Jewish mother or a convert to Judaism—the boundaries of Haredism are broader and more indistinct. Scholars therefore refer to the Haredi community as a “society with broad margins” (Leon 2010). In the course of time, some conceptual definitions have emerged. Today, first and foremost, there is an in-group definition, according to which one can be called a Haredi when 1) seriously observing all religious regulations, 2) when sending his children to institutions of the Haredi educational system and 3) when—with limitations—not performing full military service in the Israeli army.

Haredi Jews claim to fulfill all religious commandments derived from the Torah and to live according to the biblical Jewish law (Halakhah) and its strict interpretations, referring to Talmudic sages of the antiquity and to the rabbinical scholars over the last 2000 years. In order to maintain their communities, the lives of the Haredim have been devoted to entrenching their interpretation of Judaism in the face of social innovations, rules and regulations within a secular and liberal society, which are considered “Chukos haGoyim,” the “Laws of the Gentiles” (Heilman and Friedman 1994, p. 198).

Like most other modern fundamentalism movements, Haredi Judaism emerged in response to the secularization process, the Jewish Enlightenment (Haskalah), the internal Jewish reform movements (progressive and conservative Judaism), nationalism (Zionism), and socio-political emancipation that had developed among Jews in Eastern and Central Europe over the 19th and 20th centuries (Kimmerling 2004, p. 23). The origins of Haredi society in today’s Israel lie in the so-called Old Yishuv, i.e., among the Jewish population that lived in Palestine before the proto-Zionist immigration of the 1880s, as well as among (ultra-)orthodox Jews who immigrated from Europe, the Middle East, and North Africa after World War I and especially during and after the Shoah. Today, the contemporary Haredi community in Israel is divided into three main Jewish streams of different theological, cultural, and geographical imprints, which will be presented in short in the following:

### Haredi jewry I—The followers of Hasidism

The movement of Hasidism, whose representatives are called Hasids, in Hebrew Hasidim, emerged in Eastern Europe in the 18th and 19th centuries. Charismatic spiritual leaders, so-called “tsaddikim” (righteous men), formed Hasidic courts, and established Hasidic dynasties (Hasidut). The leader of a Hasidic dynasty is called “Admor,” the acronym of “Adonénu Morénu veRabbénu” (Our master, our teacher, our rabbi). The mythological founder of the Hasidic movement is considered to be Rabbi Israel ben Eli’eser (ca. 1700–1760), called the Ba’al Shem Tov (Bearer of the Good Name) or HaBescht for short, who worked in Western Ukraine, then part of the Kingdom of Poland (Talabardon 2016, p. 43). After the almost complete annihilation of the Hasidic communities in Europe during the Shoah, many Hasidic groups recovered or were reinvented. The most numerous and influential ones in the 21st century are the Hasidim of Gur, Belz, Vishnitz, Satmer, Chabad Lubavitch, and Breslav.

A modern-day Hasidic Admor strives to establish a more or less direct descent from the Ba’al Shem Tov or his disciples from the legendary first Hasidic “community of [the town of] Medshybish,” which is also related to as the *Golden Age of Hasidism*. Each dynasty therefore meticulously maintains the genealogy of the respective Hasidic “Rebbe,” which must reach back to the origins and include all generations of fathers without gaps. In addition to the study of the Torah and the Talmud, Hasids cultivate practices of Jewish mysticism, Kabbalah, asceticism, meditation and Jewish folk traditions of Eastern Europe (clothing, prayers, singing, dancing). After the almost complete annihilation of Hasidic communities during the Shoah, it is estimated that at the beginning of the 21st century there are again a few hundred different large, small and micro Hasidic groups. The Hasidic centers in Israel are Jerusalem, where dozens of Hasidic courts are located, and the city of Bnei Brak in the greater Tel Aviv area. Constant secessions, usually the result of inheritance disputes following the death of an Admor, cause the number of Hasidic courts to increase continuously. However, there is also the phenomenon of dying Hasidic micro-dynasties which unite with larger ones that are more promising and materially more potent.

### Haredi jewry II—Lithuanians/mitnagdim

The followers of yeshiva Judaism that emerged in the 19th century on the territory of Lithuania (then Russian Tsarist Empire) are grouped under the term “Lithuanians,” Hebrew Lita’im or Litwakim. They are also called “Mitnagdim” (opponents) due to their historical antagonism to Hasidism. Mitnagdim see themselves in the tradition of Rabbi Elijah Ben Salomon Salman (1720–1797), the so-called Gaon of Vilna. Salman, who was considered the leading Jewish scholar of his time. He had issued several bans and excommunications at the end of the 18th century against the Hasids, whom he considered heretics because of their reinterpretations of Judaism at that time. Hasidim and Mitnagdim reconciled twice. First, in the course of the 19th century, the wake of the “great erosion,” i.e., the mass departure of Jews from the strictly religious communities and their turning to contemporary ideologies—secularism, socialism, communism, Zionism, anarchism, etc.—which was perceived as a common threat. The second time after the Shoah, when the sheer survival of traditional religious Judaism seemed to be at stake and forces had to be combined.

In the 19th century, Lithuanian rabbis established the concept of the Great Yeshiva as a supra-congregational, “total” institution (Kimmerling 2004, p. 237). As in a Christian monastery, in a yeshiva men form a secluded society of learners who are under strict supervision and develop their own norms of behavior, which they then project onto the general public outside their own walls. In the course of the 19th and early 20th centuries, the first yeshivot and their leaderships gained an independence from the respective local Jewish community through the establishment of a complex fundraising system, with funding primarily from Jewish donors in Germany or the USA.

The Lithuanian yeshivas are elitist in nature and based on achievements in learning, excellence in knowledge, scholarship, and most importantly, the funds of the respective yeshiva leader, the Rosh Yeshiva. In the state of Israel since the 1950s, yeshivas have developed into economic institutions that also provide income to many parental homes within the ultra-Orthodox community and that live not only from donations—mainly from donors in the US—but also from considerable Israeli government funding. The emergence of such a yeshivish “Society of Learning Men” (Khevrat Lomdim), sometimes also called “Scholars’ society,” based almost exclusively on resources outside the community and on the state, created a new division of labor within Judaism with the credo “These learn Torah and preserve traditional Jewish identity, and those feed it” (Friedman 1991, p. 13). The learning men thus give legitimacy to the money-makers and, to a certain extent, to their secular lifestyle (Kimmerling 2004, p. 238).

The heads of the prestigious Lithuanian yeshivas—Ponivesh, Hevron, Mir, Slabodka and Ateret Yisrael—are considered the most authoritative decision-makers in Jewish law. After the era of the influential rabbis Elazar Menachem Schach (1899–2001), Yosef Shalom Elyashiv (1910–2012), and Aharon Leib Shteinman (1914–2017) the Lithuanian public in Israel has been undergoing a process of diversification.

### Haredi jewry III—Sephardic haredim

Ultraorthodox Jews originating from the Muslim countries of Northern Africa and the Middle East are subsumed under the collective term “Sefardic,” Hebrew Sefaradim. They have adopted the ultraorthodox lifestyle of the European-originated (Ashkenazi) Haredim in Israel. Sefardic Jews—pejoratively “Mizrahim” (Orientals)—are considered descendants of the Jews who lived on the Iberian Peninsula before the expulsion from Spain (ca. 800–1500), even though many have no genealogical connection whatsoever (Rauschenbach 2018). Between the 1950s and 1970s, for lack of their own educational institutions in Israel, children of traditional Arab, Kurdish or Persian Jews attended the privately organized, state-recognized institutions of the Ashkenazi Haredim. There they learned the Ashkenazi prayer rite, customs, dress codes and even language, Yiddish. Later, the Lithuanian yeshivas served as role models for their own Sefardic Haredi institutions that began to emerge in the 1970s.

Sephardic Haredim are considered “soft Haredi” (Leon 2010), for a lesser strict interpretation of Jewish law and for more integration into broader Jewish Israeli society. Ever since the 1980s, the majority of the population of the ultraorthodox Sefaradim are affiliated with the political movement of Shas under the spiritual leadership of the late Ovadiah Yosef (1920–2013). Under the credo “Restoring the Crown to its Past Splendor” (Lahakhsir Atarah leYoshnah) Shas has been building up its own yeshivas, educational and welfare institutions. The death of Yosef led to a diversification of the Sefardic public, with his sons still holding key positions in Sefardic ultra-Orthodoxy.

## Haredism as fundamentalism—The attempt of a definition

Each fundamentalist movement is factually unique and can only be understood in its particular historical and cultural context (Bruce 2008). As is the case with Haredi fundamentalism. Ever since the suggestion, that the term “fundamentalism” can be applied to all religions and also for non-militant groups (Armstrong 2000, p. XI), a fervent controversy has evolved. Critics reject the inflationary use of the term “fundamentalist” as a categorization criterion (Emerson and Hartman 2006, p. 130–131) and caution against applying it too liberally to religious “groups we don’t like,” similar to the term “sect.” Also with the Jewish religious movement of ultra-Orthodoxy, which suffered horribly and was almost wiped out during the Shoah, a conscientious analysis is appropriate. Since the 1990s, a large amount of study on Haredism and on the analysis of its religious fundamentalist has been done (Friedman and Shelhav 1985; Friedman 1991; Heilman and Friedman 1994; Ravitzky 1996; Katz 1997; Hakak 2004; Samet 2005; Aran 2006; Baumel 2006; Spiegel 2011; Kaplan and Stadler 2012; Fisher 2016).

In this article, I will try to demonstrate the Haredi case along the definition of fundamentalism as suggested by Pollack, Demmrich and Müller. Accordingly, religious fundamentalism can be characterized by four components: A) the claim to exclusive truth, B) the claim to superiority over all other positions, C) the claim to the universal validity of exclusive truth, and D) the demand to restore the unadulterated, submerged past by radically changing the present (Pollack, Demmrich and Müller 2022). To a greater or lesser extent, all of the four components can be found in the moulding of Jewish ultra-Orthodoxy at the end of the 20th and beginning of the 21st century.

### A. The claim to exclusive truth

The Haredi entrenchment, as is argued within the ultra-Orthodox community, has turned out to be only a successful and truthful way in preserving *Jewish continuity* and preventing acculturation and, finally, *assimilation* within the non-Jewish environment. Or, as one of the most prominent spiritual leaders of Haredi Judaism in the second half of the 20th century and head of the “Council of the Torah sages,” Rabbi Elazar Menahem Schach (1898–2001), put in a widely noticed speech in Tel Aviv in 1990: “We are the people of Israel, what are we among the nations that surround us with evil? What can we do? We (Haredim) do not just say (any) theory. I tell you true things. The people of Israel, what happened to them, exile after exile, anti-Jewish laws after anti-Jewish laws, slaughters and exterminations, burnings and murders—strong nations wanted to wipe us out. They lost. Two thousand years we are alone, empty-handed, without weapons, how can we stand up to that? And we have won. We stand there and exist. What is the secret behind this? What is the miracle of it? The miracle is that I am a Jew and I am stronger than them. Kill me, but my sons will live. I will walk on the path of my father, on the path of my grandfather, and my grandmother. […] I live and you are dead. I am alive, I will be, if I do not cut ties with my fathers. I will live if I do not seek other cunning and arrogance. […] I will learn my wisdom, my own culture. […]”^4^

In text-based traditions, fundamentalists regard a core element of their texts, which are considered sacred, as infallible and inerrant, for example the Bible or the Koran, to which (in parts) divine authorship is attributed. Monotheistic book religions are therefore particularly susceptible to fundamentalist manifestations (Assmann 2015). The role of sacred texts is elevated to the position of higher authority, and all other potential sources of knowledge and meaning are subordinated to it (Kressel 2007, p. 51–52).

Haredim consider their sacred text, the Torah, as the holiest within a range of “holy [Jewish] literature” (Sifrut Kodesh). They perpepuate the traditional Judeo-Christian religious belief, according to which at Mount Sinai the monotheistic god YHWH orally gave the Torah—the first five books of the Hebrew bible—to the Jewish prophet Moses, who then wrote it down. As of divine origin, the Torah is perceived as the most authoritative source of all values, moral, and law. As all aspects and all questions of religious, legal, social and political life shall be answered by the writings of Judaism, the Mishnah and Talmud, the scholars’ commentaries on the Torah, play *the* central role in Haredi Judaism. Other permitted works on the “Jewish bookshelf” are prayer books, books on religious legal precepts such as the Shulchan Aruch, the esoteric book of Zohar, various moral literature and writings of the respective Haredi sub-strands, for example Hasidic folk tales.

In order to maintain in-group religious authority over the exclusive truth, which ultimately always derives from the Torah, Haredi Judaism has developed the concept of “Da’at haTorah” (Opinion of the Torah). Haredism being a patriarchal religious movement, consequently only a “G’dol haTorah” (Master of the Torah), i.e., a male rabbi or scribe who is dedicated to religious study with or without affiliation to a yeshiva, is bestowed the ability to judge in a matter in a life of a Haredi Jew (Braun 2005). Following the rules and regulations of the “Masters” is considered the only legitimate and righteous path in preserving authentic Judaism and in the struggle between good and evil. In each generation, the honorary title “G’dol haTorah” and the related religious authority is given to an approximately dozen leaders within the Lithuanian, Hasidic and Sephardic communities. Over the last decades and following major divisions and split ups, each major Haredi community has established its own “Council of Torah Sages,” which prescribes the matters of their communities and the actions and voting behavior of its political representatives (Braun and Leon 2017).

As the Haredi community considers itself under constant threat, a central means of keeping the non-Haredi society off limits and of controlling its own society is the pursuing of the ancient biblical concept of “purity” and “impurity.” The demarcation occurs through the excessive preoccupation with the bodies of the community’s members (Douglas 1966) by meticulous observance of food taboos (Kashrut), touching prohibitions (Niddah), and clothing regulations (chastity). By these regulations, the boundaries between the private and the political body become blurred, and the Haredi individual eventually loses autonomy over it (Aran 2006, p. 89).

Non-Jewish sources, the so-called “Chukos haGoyim” (Laws of the Gentiles), are considered untruthful and dangerous, destroying Judaism and the world as such. Therefore, Haredi society has imposed strict censorship. Freedom of opinion and freedom of information are suppressed. In a Haredi household, there are (officially) no “impure” books or newspapers. Writings that do not conform to the canon of the acceptable “Holy Literature,” like scientific or reformist Jewish works, are forbidden. Modern means of technology and communication—for being potential gateways for the process of secularization—are meticulously scrutinized for they might harm the information policy within the community.

The pashkvil, a printed poster in archaic and alarmist language with a narrow typeface and without pictorial or graphic elements, is the only all-Haredi permissible medium for daily communication. It continues to form an informal and anarchic information system also in the 21st century in the Haredi neighbourhoods in Israel. Here, rabbinical authorities publish their judgments, families announce obituaries, and anonymous authors disseminate news, gossip, criticism and slander (Levy 1990). Even though, the home computer, the internet and new telephone technology have been forming a serious threat to the walls of Haredi entranchment, the communities have found ways to adapt them, e.g. by using “kosher cell phones” without internet access, data, video, or messenger services as a widely accepted norm (though in reality it is circumvented by at least one third of the Haredi public). The Covid-19-pandemic, which hit the Haredi community hard, accelerated the use of internet also in the Haredi society (Malach and Cahaner 2021).

As in other puritan fundamentalist societies, that simplify complex contexts, the Haredim consider the body to be the source of the “evil inclination,” in Judaism called Yetzer haRa. They therefore devote disproportionately high attention to both ritual and sexual purity. Both sexes must dress in such a way, that the sexual drive is not provoked. The clothing is an inseparable part of a Haredi man’s identity, a uniform that signals his belonging, and it is even considered to have miraculous effects. Also language itself is a characteristic for the demarcation mechanisms of the Haredi population between “pure” and “impure.” The language of the Bible, Ancient Hebrew, and rabbinical Hebrew are considered “Loshn Koydesh,” the Holy Language, which shall not be distorted by profane use. Though a majority of the Haredi Jews in Israel speak Modern Hebrew, they use distinguished expressions in order to distance oneself from secularized Hebrew. Yiddish, the traditional language of the Eastern European Jews, which is considered the “holy language of the Martyrs of the Shoah,” is only preserved by strictly anti-Zionist Haredim, like the Satmer Hasidim, who use it in daily routine and education (Baumel 2006, p. 96).

Noncompliance with the above mentioned dietary, haptic and dress codes are considered “ritually impure.” Haredi Judaism also distinguishes “moral impurity,” which includes the offenses of murder, adultery, sacrilege, idolatry, spirit worship, incest, rape, homosexuality, sodomy, and human sacrifice (Gen 34:5, 13, 27; Lev 18, 19, 20:3; Num 5, 35:35; Dt 21:23, 24:4; Ez 8:6, 18:11, 20:18, 22:11; Jr 2:23, 7:30, 32:34). In cases that are also classified as felonies by the state legislature, the Haredi community cooperates with the state authorities. But cases of corruption, white-collar crime and sex crimes are hushed up by the Haredi community and the perpetrators often escape conviction by the civil state courts, similar to the institutional cover-up within the Catholic Church.

### B. The claim to superiority over all other positions

The essential element that unites the contra-acculturationists is the confidence that Jewish life and tradition is an alternative superior to anything non-Jewish contemporary culture could offer (Heilman and Friedman 1994, p. 213). Therefore, Haredi Judaism denounces other religious Jewish denominations and secular Jewish identities, which in their eyes acculturate with their non-Jewish environment and therefore undermine the Jewish future by uprooting the past.

Rabbi Elazar Menahem Schach put this claim against other Jewish identities—especially the secular Zionists—in his famous speech back in 1990: “You are also a Jew, you are circumcised. But do you know your own culture? And what is your culture? The English one? That is your culture? … When you open the Torah, do you know the meaning of the words? […] There are those, who say, that the words I am saying, they are the words of a fanatic. Old things. But the Holy Torah says “Surely, this is the wisdom and understanding in the sight of the peoples” and this testifies that the Torah is the wisdom of the Jews in the eyes of the nations.”^5^

The Haredim are convinced that they possess the ultimate truth and that in the end they will triumph over the public space in Jewish society. They are guided in this by the conviction that the currently prevailing alternative to their own lifestyle, secularism, is doomed to extinction, however seductive it may be to members of the Haredi community and however dangerous it may be to their own way of life. All true believers must therefore protect themselves from being contaminated by the secular lifestyle (Heilman 2005). As of the early 2020s, due to the the demographic developments inside the Jewish world, some Haredi speakers even talk of the “Haredi momentum” which is winding up, winning over the public sphere (Spitzer 2022).

Even though closely related to each other over transnational bondings linkages, Israeli ultra-Orthodoxy also has been suspecting the American Haredi strands of too much acculturation within the non-Jewish sphere. The reason for this can be found in the active counter-acculturationists claim after the Shoah, that the only legitimate way to prevent assimilation is the relentless fight against all outside influences, including the host society, and not allowing oneselves to be assimilated by the modern world even in the quest for funds for their institutions. Only the ongoing struggle against everything that led to acculturation could keep one a true Haredi (Heilman and Friedman 1994, p. 221–222).

### C. The claim to the universal validity of exclusive truth

Haredism—no more and no less—considers itself as preserver of the universe. Haredi Jews interprete the biblical phrase from the Book of Jeremiah 33:25 “This is what the LORD says: If I have not established My covenant with the day and the night and the fixed order of heaven and earth” as such: “If my covenant,” i.e. the Torah, “is not implemented day and night,” i.e. by the continuous study of Torah, “there is no heaven and no earth,” i.e. there is no existence of the world. Therefore, Haredi rabbis, scholars and speakers of the community regularly use the phrases “Without the study of Torah, there is no existence on earth” or “Without Torah there is no ethics, and without ethics there is no world,” referring to the Torah as being the ultimate source of all values, moral, and law.

For example, when speaking to the Haredi leadership in Israel in summer 2013, the Lithuanian Rabbi Aharon Leib Shteinman declared (Shteinman 2013): “The Lord made that the Torah is man’s life. Without Torah, God beware, there is no existence. And because not everybody is capable of studying all day long, the Lord made, that one man can study and the other is providing for him. One has to know, that all the life of man is Torah. Without the Torah, the Whole of Israel and the world does not exist. The whole creation is based on the Torah” (Lev 2013). Refering to the biblical creation myths (Gen 1:1–2:3) the universal validity is based on the alleged sacredness of the Torah and its divine origin.

### D. The demand to restore the unadulterated, submerged past by radically changing the present

Where religion has lost its social and political hegemony, fundamentalists have developed their own segregated cultural, geographic, and religious enclaves in which they lead and maintain their ideal lifestyles and consider those who do not practice these forms as not belonging (Almond, Appleby, and Sivan 1999, p. 92–93; Eisenstadt 1998). Similar to other fundamentalist religiosities, in order to relieve the tension of resisting the modernity on which one depends after all, Haredi Judaism turned inward in the beginning: The focus was on strengthening the faith and religious foundations of its members, insulating them within a cultural enclave from the undesirable effects of modernity. The co-founder of modern Jewish ultra-Orthodoxy, Moses Schreiber (1762–1839), called after his pen-name Chatam Sofer, coined the future tenet for Haredi Judaism borrowed from the Talmud: “The new is forbidden according to the Torah” (Samet 2005). Ever since, Haredi Judaism sees the essence of Judaism in the communal, segregated lifestyle that the hostile environment had imposed on Jews before the age of emancipation, and it considers the embodiment of Judaism in the performance of practices that had been accepted as obligatory in pre-Enlightenment times. Heilman and Friedman considered this phase of counter-acculturation as *passive fundamentalism* (Heilman and Friedman 1994).

Things changed after the Shoah and after the establishment of the state of Israel. The true Haredim cannot be content to be simply passive in their traditionalism; they must be active in their *Kulturkampf*, ever demonstrating the religious “truth” to the majority who are enticed by the hedonistic pleasures of contemporary culture and who thus submit to desire (Heilman and Friedman 1994, p. 216). While secular and religious Zionism as well as progressive Jewish religious currents try to reconcile particularistic Jewish identity with concepts of modernity, e.g. nationalism and nation-state, civil society, democracy, or human rights, and allowing the study of concepts outside the Jewish religious tradition, Haredi Judaism denounces these concepts. But still, since 1948, the attitude of Haredi Jews in Israel over the last seven decades has shifted from isolationism towards certain political activism and cooperation with those protagonists in the Jewish realm, whom they consider most supportive for their own lifestyle.

In the phase directly after the Shoah, i.e. the near extinction of Haredi Jewry, several changes within Israeli Haredi society and its political and social participation can be observed. Between the years 1948 and 1953, after the victory of Israel in the war against the Arab population and the establishment of the new state, Haredi Jews were of enthusiastic and cooperated on a large scale, e.g. in the army and in politics. From 1953 onwards until the late 1970s, Israeli Haredi society actively isolated itself from the rest of Jewish society, which it then considered a serious threat for its own survival. The main means in order to change the allegedly perilous present was the re-construction of the Haredi educational system, the building of local religious structures and of Kashrut-surveillances all over the young state. Decisive for this development was the Lithuanian Rabbi Avraham Jeshayahu Karelitz (1878–1953), called the Hazon Ish (Braun 2011), who initiated this trend. This was also the period, when the new “Society of Learning Men” was evolving and flourishing, enabled by public financiation from the welfare state.

Counter-acculturative activists are obsessed with the control of sexuality because that is one of the most attractive aspects of modern life (Heilman and Friedman 1994, p. 217). Therefore, in the period of turning inward and rebuilding the community, Haredi Judaism focussed on repressing and redefining libidinal pleasure as iniquity, which powers the modern world (Ibid). Over the last seven decades, the female body in Haredi society has been gradually sexualized and women’s basic rights within the Haredi community have been severely curtailed (Moller Okin 1999). For blood is considered “impure,” Haredi women over the time of their monthly period and after giving birth are not allowed to be touched by their husbands. These Niddah-regulations shape a strict regime of the women’s and the Haredi couples’ sexuality. In the public sphere, the Haredim practice a strict separation of the sexes: touches between men and women (who are not members of the family) are forbidden and they have to keep certain distance, in the synagogues women must be seated on a separate gallery, where they cannot be identified by the men. In public transport in Haredi strongholds in Israel, Haredi men and women sit in separate areas—men in front, women in the rear. Women are not entitled to represent the Haredi community in public. Even women’s voices are sexualized and literally silenced by the prohibition based on the Talmud saying “The voice of the woman is shame” (BT Brakhot 24). Haredi women are not allowed to sing in the presence of men who are not members of the immediate family. Women are forbidden to sing for mixed audiences in public, such as in concerts. And, they are not allowed to present the Haredi public as politicians in local or national politics.

Once a fundamentalist movement has gained stability and self-confidence, it takes on political activism with the goal of restoring the influence of religion (Juergensmeyer 2008). The walls of the enclave are thus becoming more permeable. Instead of being satisfied with religious self-improvement, the fundamentalist movement is now trying to expand its influence externally in various ways, through social services, religious preaching, political participation and, if necessary, violence (Sadowski 2006).

In the Haredi case, this development can be seen since the late 1970s. With the end of social-democratic Zionism and its ideology of the “New Hebrew Man” since the late 1970s—which Haredi leaders considered as anti-Jewish at its core—Haredi participation in public Jewish life began to gradually increase. First, in the 1980s and 1990s by limited cooperation in governments with both right- and left-wing Zionist parties—first and foremost in order to secure the material means for the ever growing Haredi community and for its educational system. Later, in the 2000s and 2010s, after a process of diversification within the Haredi camp, most prominently the Sephardic educational system and the Shas-party, the Haredi political representatives started working closely together with right-wing and national-religious acteurs in Israeli governments. Now, in the early 21st century, Haredi politics started actively cooperating in the fight of the non-liberal Zionist camp to diminish the democratic aspects in the Israeli legislation. What seemed unlikely 20 years earlier, Haredi parties now supported laws against human and civil rights organisations, against the Arab minority in Israel, against the Palestinian population in the Occupied Territories, against the freedom of speech and initiated laws against liberal Jewish denominations in Israel (Dödtmann 2021, p. 162–176). Being part of most Israeli governments between the years 2009 and 2021, they also thwarted attempts of the liberal opposition to oblige the Haredi educational system to provide a scientific education for its pupils, attempts to oblige all of the Haredi male public to serve in the Israeli army and attempts to ease the regulations for conversions to Judaism and for the definition of Jewishness.

In the process of increasing participation in Jewish Israeli society, the Haredi leadership and the communities’ members face the influences of modernity. Since the 2000s, a population stratum of a working Haredi middle-class has developed. They exercize certain behaviour which some decades ago was considered strictly taboo, but now is accepted—most prominently certain consumption and leisure activities such as travelling inside Israel and abroad, Haredi women watching movies made by Haredi women (Vinig 2021), and even some sports and arts activities when in the frame of gender segregation and chastity. In general, arts in the Haredi society like music, theatre, cinema, and painting all have to be related to the Jewish faith and to portray the Jewish Haredi way of life uncritically.

Facing this exploring of boundaries, the Haredi leadership is expressing the urge to continuously “strengthen” (lehitkhasek) the community’s members in their belief and lifestyle within the Haredi realm. For those, who are not able to repress their (libidinal) desires, which modernity implicitly or explicitly offers, are considered “weak” and the threat for the future of the ultra-Orthodox Jewish continuity. Thus, as already Heilman and Friedman suggested, fundamentalism in the Haredi case can be considered as a psychological rather than as just a social conflict (Heilman and Friedman 1994, p. 217).

In the following there will be examined, to what degree the *Status quo* in Israel is constitutive, supportive in perpetuating and, presumably, even amplifying the religious fundamentalism moulding of Haredism in Israel. For this, the aspects of educational autonomy, its implications for the labour market, the exemption from military service, the judicial autonomy and hegemony in personal matters of a Jew, as well as the combat over the Shabbat as the holy day of rest in Jewish Israel will be discussed.

## The autonomous Haredi education

In fundamentalist hands, the yeshiva is no longer simply a place for scholars: it becomes a frontline battle station in the war against “Chukos haGoyim.” As such, not only Mitnagdim need them, but also Hasidim and Sefaradim (Heilman and Friedman 1994, p. 216). In order to preserve its isolationalist lifestyle, Haredi Judaism is keen to further develop and increase its educational instutions—as means as of closely binding its members and as a place to reinvent Jewish tradition.

Education in Haredi society is not considered an individual, general and social right, but a religious duty. Or, as the Lithuanian rabbi Aharon Leib Shteinman put it in a rabbinical op-ed in his factional newspaper *Yeted Ne’eman*, Torah study is the very reason for the existence on earth: “The goal of creation is Torah. Of anyone who has thought otherwise in all these generations, no memory remains” (Yeted Ne’eman 2008). Learning the Torah is a mitzvah, a biblical duty, a religious act supported by society. And, as is the case in a patriarchal society, the male, who is the sole carrier of the “pure” sphere of spirituality, gets religious education and upbringing for shaping him into an idealized image of a strictly religious Jew, faithful to the religious laws. In the Haredi educational system, the boy learns the codes of its society: to observe the Torah, the biblical commandments and prohibitions, the sense of Haredi *Jewish continuity*, and the sense of righteousness, social stability and perfection against the whirlpool of the modern world (Spiegel 2011). As a “Servant of God,” a Haredi Jew is not to be concerned with self-development for its own sake, but with fulfilling the will of his Lord. The fulfilment of God’s command and the observance of his commandments are thought to be the true self-realization.

Haredi education is referred to as “pure,” following the “proper norms” of Haredi society and it is off criticism. Haredi schools are seen as part of the “sacred sphere,” separated from the profane, impure world. Here, the “children learn in the rabbi’s house,” as opposed to the “children, who have been captured by the gentiles,” i.e., who do not receive an education in Jewish tradition and Jewish law. The Sephardic rabbi Ovadiah Josef warned its community against secular schools: “Do not send your children where there are secular teachers who do not know Jewish law. The secular teachers are not teachers; they are donkeys” (Ben Haim 2018). (Ovadiah Joseph’s omission is based on the similar sound of the Hebrew terms for “the teachers,” HaMorím, and “donkeys,” Khamorím.).

The Haredi school system cements gender segregation, with boys and girls attending separate institutions from the age of three. While girls receive a minimum of general education and vocational training in order to gain a foothold in the labour market, boys are prevented from attaining an adequate education, that would allow them to pursue a professional development outside the ultraorthodox world. The boy is confronted exclusively with material that negates the values of a modern, secular, liberal society. Haredi Jewish learning should not help a boy gaining knowledge or vocational training, but rather functions as a means of maintaining and deepening the cultural insularity of the community. It is considered the main tool to preserve the Jewish tradition.

Tools of fundamentalist Jewish education are, 1) long days of learning and little free time, 2) teaching largely “holy” subjects, 3) using traditional teaching methods with largely religious scriptures, 4) the lack of a curriculum coordinated with the state education system, 5) teaching by teachers who have not undergone academic or pedagogical training. The Haredi boy is supposed to internalize, at best memorize, the Jewish law, its analysis and interpretation, the Gemarrah. This is his “ticket of entry” into the closed Haredi society and a prerequisite for a favourable match on the Haredi marriage market. Therefore, a Haredi family is ready to sacrifice itself for the boy’s education, to fully support him ideally and materially. However, admission to the desired and considerably best institutions depends on many factors: the ethnic profile (Ashkenazi “white” Haredim are preferred), the status and name of the family within the community, membership in an ancestral list of rabbis, the degree of strict religiosity, or the willingness to donate to a yeshiva (Dödtmann 2021, p. 231).

Each Haredi subgroup is constantly striving to establish and expand its own educational institutions, which compete in the “open Haredi educational market” (Spiegel 2011, p. 102). Because of the ceaseless efforts in institution building, the Haredi community is also referred to as a “society of institutions” (Friedman and Shelhav 1985). By the arrangement of the Status quo, the Haredi educational system enjoys large autonomy. It is the fastest growing branch within the fragmented educational system of Israel. From approximately 212,000 children and young people in the year 2000, who were studying at Haredi institutions, the number has risen to more than 404,000 in 2015. Thus, nearly one in four pupils in Israel attends the Haredi preschool and school system. As the vast majority of Haredi schools are private and decentralized, they are financed by fees and donations as well as by public funds—up to 100% of the school’s budget—from the state and from local authorities. Therefore, they are supposed to be subject to legally established controls. But, the Ministry of Education does not adequately fulfil its role as a supervisory body, it interprets statistics to its own advantage and distributes funds to the various Jewish interest groups on a scattergun basis. As a consequence, opaque nepotism and corruption are typical of the Haredi institutions (Dödtmann 2021).

In most schools for boys between the age 5 and 13, 55 to 75% of the so-called Tokhnit Liba (basic program) of government schools, i.e. secular-scientific content, must be taught. Since the requirements are not consistently controlled, the proportion is lower, and often the boys do not learn English at all. All areas of knowledge in the secular subjects are embedded in the religious context; the history of other countries and peoples is always read in the context of relations with Judaism. Science is considered speculation, as opposed to absolute truth, i.e., the certainties expressed in the Torah. The norms of society are reflected throughout the textbooks, e.g. by the lack of photographic depictions of women. Geography and history are treated from the perspective of the Land of Israel. Though otherness is tolerated in Haredi textbooks, it is not recognized as equal to that of (Haredi) Jews and presented as a potential threat. Democracy, civil society, the emancipation of women, nationalism and liberal religious currents in Judaism are rejected. Religious and racial stereotypes toward others, especially the Palestinian neighbours are not dismantled (Pardo and Gamliel 2017).

The most decisive stage in Haredi male education starts after the Bar Mitzvah, the Jewish rite of passage for adulthood, at age 13. Then the Haredi boys transfer to the Minor Yeshiva and later on, from the age of 17 until marriage, to the Greater Yeshiva (Shiffer 1999). In a yeshiva, which is often designed as a boarding school, the boys and young men experience a closed community of full-fetched surveillance, comparable to a “total institution” (Stadler 2009; Goffman 1961). The yeshiva is idealized for being the perfect sphere of “purity.” It is the only place where the ideal of a Ben Torah—a Jewish man fully devoted to religious study—can be realized without concern for economic needs. Here, a student’s religious personality is formed at a time in his life when his non-Haredi Israeli peer is preparing for a profession or for academic training. In a yeshiva, Talmud and Gemarrah is studied exclusively, from 7 in the morning until 8 or even 10 in the evening. Supervisors, so-called “mashgikhim,” “morally instruct” and disciplinarily punish the pupils in a yeshiva, e.g. for dressing inappropriately, for using non-kosher cell phones, or for having illicit contact with girls. After marriage, a Haredi man—as long as his family can afford it—continues learning at a kollel. The kollel offers young husbands a framework adapted to their life situation and where questions about family life are addressed. This institution provides a prolonged influence on the young man’s personality. The kollel as an institutional continuation of the Great Yeshiva had become necessary, as the average age of marriage for young Haredi men dropped to 21.

A boy’s journey in the Haredi education is supposed to be life-long, but in most cases lasts an average of 20 to 25 years. Usually, rabbinical authorities do not permit the young man to take up vocational training or engage in regular gainful employment before the age of 24. Only after the young man has married and become a father is he considered to be so deeply socialized in the community and economically dependent on his strict religious environment that leaving this way of life has become unlikely. However, in their thirties at the latest, the majority of Haredi men for financial reasons take up employment: in the Haredi educational system, in areas of religious life, or in secular professions. A small group of outstanding or well-connected yeshiva graduates make careers as religious judges (dayanim) in the private and state rabbinical courts or take up the lucrative profession as private or state Kashrut-supervisors.

By Israeli law, a yeshiva or kollel student from the age of 18 is required to serve in military service. But, Haredi students can defer from it by help of the regulation of “Toratam Omanutam” (Their Torah is their profession), and after marriage and the birth of their first children, they are only drafted into the army for a shorter period or not at all. Still, the country’s militaristic discourse seeps into the Haredi realm of education (Stadler 2009). Yeshiva students see themselves as “soldiers” and protectors for the People of Israel and therefore as an integral part of the nation’s defence (Zikherman 2014, p. 320). They imitate the model of the military world, which is inaccessible to them, through extreme physical discipline and mortification during their Talmudic training. Learning in an ascetic and total institution and the associated constant struggle against the Yetzer haRa, i.e., the sexual drive, is described by the religious students as an even more difficult task than that of being a soldier.

Since no state high school diploma can be earned at Haredi yeshivas and since the majority of the rabbinic leadership rejects military service, the yeshiva system is the only legal framework for young men to make a modest living in the framework of the “Society of Learning Men.” They have been supported by a wide range of social benefits such as government grants, donations, income support for families with three or more children, or affordable housing. These benefits and state support have been provided by the political arm of the ultraorthodox community, the Haredi politicians when in the Israeli government, which is the case for most of the time since 1977. The moment the young man leaves the Yeshiva or the Kollel, the payments cease.

## Haredim and the labour market

While men in modern society are defined by the constraints of the economic world, the Haredi model of pious masculinity strives not for professional success but for excellence in the world of yeshivas as the highest value. Everyday business is considered inappropriate for the Haredi man, who strives for piety and lifelong study of the Torah and its interpretations—the man’s “realm of the holy” (Stadler 2009, p. 74). Work, investment, and commerce are relegated to the realm of physicality and are considered earthen and profane. The world of work is seen as harmful and “impure.” A workplace is seen as a “jungle,” a place “of aggressiveness, fuelled by sin and transgression” (Kimmel 1996, p. 55).

The influential Lithuanian rabbi Elazar Menachem Schach once summarized the Haredi ideology towards work as such: “According to the gentiles, […] man was created for the world, he must open it up, improve it […], fly to the heavens and the result of this is: the good of mankind! The cycle is the following: I must form the world to take from it, and if I take from the world, then I form it further […]. A repeating cycle, without any purpose, without any goal! […] Those who, according to their conception […] can be happy only when they “take” from the world and shape it for their use and for their convenience […] for them we can feel only pity” (Hakak 2004). In 2007, Schach’s successor Aharon Leib Shteinman called professional work a “poison” and publicly spoke out against vocational training for young Haredi men (Ettinger 2007). In order to attain perfect godliness and maintain their position as an elite, Haredi men must detach themselves from the material world and from work, which is considered a punishment imposed on mankind since the expulsion of Adam and Eve from Paradise. Even if a Haredi man finds himself in financial difficulties, he should study Torah intensively. Thus, a Haredi man experiences is a permanent tension between “faith” and “effort” in his life (Kay 2012, p. 168).

Work in Haredi ideology is not an option for personal aspirations and has no value unless it is for the health of the body and soul, to overcome the “evil drive” or for the necessary subsistence. In the ascetic model of a religious scholar, transcendental rewards are promised for the economic difficulties and poverty stemming from the rejection of work: strong bonds within the community, salvation, and a sense of continuity of orthodox Judaism. This stance towards work corresponds with the theory, that “fundamentalist groups adopt ascetic practices and withdraw from society to confront and reject the assumptions and products of late capitalism” (Iannaccone 1998, p. 1465; Kuran 2004). While traditionally, rabbis saw work as an integral part of life and a place where Jewish and ethical concerns could be realized, Haredi leadership emphasizes marginal interpretations of the Jewish texts in its rejection of labour (Eyali 1987). The religious Haredi elite uses hierarchy and power to reinforce holiness and to alienate the transcendental from the economic and the modern realm of entrepreneurship (Stadler 2009, p. 75). It has also created a special language for this purpose. Haredim use the term “parnassah” when speaking of gainful employment. On the contrary, “Avodah” has a strong religious connotation of worship, as “Work in the heart” (Avodah she baLev). “Parnassah” is considered to be toil, something of a mundane nature. It cannot be compared to the artistic, divine, and transcendental characteristics of “Work in the heart” or worship of the Divine.

However, the relationship to work both on the part of the elites and at the grassroots of Haredi society has been changing since early 21st century. The rejection of capitalism and the modern work ethic, preached for nearly half a century, has been increasingly criticized internally by yeshiva students themselves (Stadler 2007). Students who cannot fulfil the ideal of a Ben Torah but have limited employment opportunities complain of frustration and existential angst. Some of them rebel against the ethic of complete withdrawal from the “profane” labour market, and argue for a new form of piety that combines religious study with livelihood and integration into the labour market of the modern state, comparable to the approach of religios Zionists. They refer to traditional Eastern European Jewish working class, which was respected by its work, donations and support of the truly learned, and helping the weak in society. To be sure, the young men accept the official view that they live in a historically unique time—after the destruction of the Torah world during the Shoah and being surrounded by a quasi-secular Jewish society—and that this circumstance demands a new form of religious devotion (Stadler 2007). A survey found that between 25 and 30% did not see their future in yeshiva or kollel, but preferring to pursue a profession (Lupo and Malachi 2012, p. 49). Haredi media are also part of the slow paradigm shift and regularly publish positive articles about the Haredi world of work. In the 2010s, a new social stratum of “working Haredi men” emerged in Israel (Zikherman and Cahaner 2012).

However, the processes of responding to growing economic pressures and internal criticism continue to be hindered by the Haredi elites. Yeshiva leaders oppose the integration of general and vocational education into yeshiva curricula. Periodically, the Ashkenazi Council of Torah Sages publishes a “Call to Holiness,” wherein the boys are reminded to “pure” study of the “Holy Torah” in the “Holy Yeshiva” as was “customary in the previous generation,” for the study of the Torah and the Jewish law are “the basis for the way of life of a Jew” (HaMevasser 2018).

Whereas in modern capitalist societies well-paid, prestigious work is predominantly performed by men and low-paid or unpaid work by women (Ortner 1996, p. 61), in Israel’s Haredi society unpaid men’s work takes priority over the hegemonic capitalist value of wage labour. Women are seen as inherently worldly and practical, and they are expected to support the family and the man’s lifelong religious studies. They are considered responsible for all earthly needs: both household and breadwinning. Although women are entrusted with these earthly affairs, they remain under strict control of the male Haredi society (Blumen 2002). In 2021, 78% of Haredi women were employed, in comparison to only 52% of Haredi men officially working (Cahaner and Malach 2021). Many of these women work in jobs outside the community, such as in IT or real estate management, where they are exposed to modern values of work and femininity that run counter to the values of their community: that of privacy, and that of compartmentalized society, which confronts them with the modern, capitalist definition of work and its gender divisions (Blumen 2002). This is rather unusual for ultraconservative groups (Griffith 1997). Prerequisite for this constellation was the establishment of the state of Israel, its welfare system, and the (partly) state-funded Haredi educational system. The establishment of the Haredi girls’ schools, the Beit Yaakov, and the seminaries for vocational training since the 1950s was followed by a growing need for female teachers within this system. Young female Haredi teachers were the perfect match for yeshiva students: they could provide for the family and remain under the control of the community (Sheleg 2000). The constellation became standard and caused the average age of marriage to drop noticeably: from the late twenties in the 1950s to early twenties in the 1970s (Friedman 1991). Once the job market for teachers was saturated, women took alternative jobs—in child rearing and care, as graphic designers or clerks—first within the Haredi community, then outside it. The professions are regularly adapted to the needs of the (all-Israeli) labour market in order to enable young women to find employment. The introduction of new professions at a seminary is previously reviewed and approved by an all-male rabbinical council of the respective stream. In this process, the heads of the yeshivas, directly determine the educational opportunities for women (Dödtmann 2021, p. 245).

The resurgence of the working Haredi wife also led to a modification and reordering of social principles and the reconstruction of femininity. Still, ultraorthodox women do not yet possess enough political power to bring about comprehensive social change. Ambitions, to represent themselves in public through party politics, have been thwarted by the rabbinic leadership and by the male-dominated society. The immediate response to complaints about women’s multiple burdens as mothers, housewives and providers is often consumption and leisure. One rather new channel for feminist criticism is the emerging female Haredi filmmaking scene (Vinig 2021).

## The exemption from military service

The state of Israel, as an occupying force which is at war with the Palestinians and which has no peace treaty with neighbouring countries like Syria, Lebanon, or Iraq, has a dire need to maintain a conscription army. The military experience and the *military mind-set* are an integral part of Jewish-Israeli identity (Kimmerling 1993). According to the *Law on Military Service* from 1949, Jewish Israelis at the age of 18 have to do military service in the Israeli Defence Force (IDF). Also, women are in principle liable for army duty, unless they are married, mothers, pregnant, or physically-mentally unfit for the army. Haredi authorities generally oppose military service for Jewish women. A “daughter of Israel” should 1) not be subjected to unsuitable conditions, i.e., the lack of gender segregation in the army, 2) she should not carry a weapon, since this is forbidden according to the Torah, 3) she is not to be confronted with the cultural norms and biographies of the non-Haredi society which are “harmful” to her own educational practice (Zikherman 2014, p. 318). In the early 1950s, ultraorthodox rabbis and their political representatives fought for the regulation to exempt Haredi women from military service for “reasons of religious family lifestyle.” Most young women from Haredi families have used this justification to circumvent compulsory military service (Cohen 2013). In general, there is no broad discussion in Israel whether to integrate these girls and women into the army.

The case is more complicated with Haredi men. In contrast to the religious Zionist Jews, who regard the state and the Zionist movement as a sanctuary and who have found a connection between army service and religious learning, Haredi authorities officially reject any military or alternative service for their young men to this day, and insist on the duty to religious learning. Anything else is considered “Bittúl Torah,” the breaking the commandment to study the Torah. In fact, an exemption for Haredi men was achieved as early as in March 1948. The military commanders of the Zionist armed forces, David Ben-Gurion (1886–1973) and Yisrael Galili (1911–1986), issued a temporary order exempt strict religious men from military service on the basis of “Toratam Omanutam” (Their Torah is their profession). In 1951, the ruling was codified in an administrative regulation. It says that a deferment of enlistment has been possible if a yeshiva student pursues religious studies full time, i.e., 45 h a week, and if he is not engaged in any other professional occupation. The exemption must be applied for anew each year to the Ministry of Defence, and from the age of 26, the person concerned can be exempted from military service altogether.

Still, the rapid demographic development of Haredi society means that not all young men can and want to live up to the ideal of the religious scholar. Since the 1980s, the Haredi authorities have been observing the problem of young Haredi men “on the street,” the so-called “shabábnikim.” While being enrolled in a yeshiva, they do not attend there and make ends meet by state allowances for religious students and through odd jobs. Because of their erratic lifestyles, Haredi society fears losing the young men altogether. Some rabbinical authorities have given their tacit consent to educate such young men in a military context to become law-abiding Jews. The IDF offers special army service for this purpose, combining military, Torah study and vocational training. In special units, which are under the strict supervision of orthodox rabbis, strict kosher cuisine is served, gender segregation is preserved, and daily prayer practices and Jewish holidays are meticulously kept.

As early as the late 1950s, there were Haredi men showing interest in serving in the army instead of learning in the yeshiva. In 1960, the first Haredi unity, the Nákhal Haredi, was formed. In the wake of political debates over Haredi participation in military service, the battalion Nétzakh Yehúda (Eternity of Judea) was established in 1999, training Haredi men to become fighting soldiers. The formation of the battalion was supported by part of the Council of Torah Sages and by several Lithuanian yeshiva leaders, who wrote in a blessing letter: “Before us was spread out the “rescue plan” for those young men who left the yeshivas whose profession is not Torah […] and because we know that their [the initiators] pure intention is to increase the glory of God through their important activity of saving these boys from descending into the pit of destruction [and] returning them to the world of yeshivas, we have come to strengthen their hands so that Divinity may enter into the deeds of their hands” (Cohen 2016). Within the next two decades, some of the young ultraorthodox conscripts also operated in task forces in the Occupied Palestinian Territories. After reports of mistreatment of Palestinians by soldiers from this unit, officials called for imposing light sentences on the perpetrators in such cases, so as not to scare away potential new recruits from the Haredi sector (Dödtmann 2021, p. 296).

While the long-time leader of the Lithuanians in the late 20th century, rabbi Elazar Menachem Schach, consistently rejected any attempt to reach a binding agreement with the state and the army on the recruitment of Haredi out of fear that an official arrangement would soon be followed by conscription orders for all other yeshiva students, Schach’s successors, Joseph Shalom Elyashiv (1910–2012) and Aharon Leib Shteinman (1914–2017), tacitly approved of the recruitment of young men into the Haredi battalion, but declined to comment on it publicly. However, they were to reap fierce opposition from numerous yeshivas and from more radical circles. The controversy directly affected, and continues to affect today, the young Haredi men serving in the IDF. They are caught in a dilemma: professional prospects and integration into Israeli majority society on the one hand, exclusion in the family environment and hostility on the Haredi street on the other. Since the army service is considered a spiritual descent, the chances of an advantageous marriage on the Haredi marriage market are reduced for both the recruit and his siblings. Some families disown their conscript sons. Other families require their serving sons not to wear army uniforms when visiting home, so as not to compromise their environment. However, the information channels in Haredi society do not allow for anonymity (Dödtmann 2021, p. 298).

In the second half of the 2010s, the IDF reported steadily rising numbers of Haredi men performing military service—between 2000 and 3000 annually. As research revealed, these figures had been falsified by army circles and were as much as two-thirds lower. Many of the recruits classified as “ultraorthodox” were in fact part of the religious Zionist segment. In this way, the army leadership wanted to fulfil the goals formulated for it—namely a growing number of Haredi recruits—“on paper.” Despite everything, as the total number of Haredi men in the conscript age cohorts slowly increases, the integration of the Haredi men poses a major challenge for the IDF: Does it want to be a predominantly secular army for both sexes or become a Jewish male-only religious army?

## Haredi influence inside the state religious courts in Israel

For conservative and fundamentalist forms of religions, the regulation of marital status and the control of family relations of its religious members with the help of religious law is an important component, in some cases even crucial to the cultural survival of the community (Hacker 2011/12). Religious personal status law is an area in which patriarchal cultures assert their autonomy at the expense of fundamental rights of the individual and of the freedom of women (Moller Okin 1999). As in many faith communities, family law in Judaism is discriminatory towards women, same-sex couples, and members of other religions and groups (Shohetman 1995). The patriarchal system of ancient Judaism has established a number of marriage prohibitions which Haredi Judaism has preserved. According to the biblical prohibition of homosexuality, same-sex marriage is not allowed in Jewish law (Lev 20:13). Furthermore, there exists a plethora of further marriage restrictions, on a wider scale for women than for men (Homolka 2009, p. 42–59). The strict marital laws ought to prevent the birth of children of “illegitimate” marriages. Children of such matrimonies are called “mamserim” (bastards) and cannot marry with halakhically “flawless” Jews.

In contrast to Western states governed by the rule of law, Israel dispensed with a uniform, all-encompassing civil judicial system and established two competing state legal systems. Matters of personal status were removed from the civil courts and handed over to nine religious courts—Jewish, Muslim, Christian, and Bahai confessions. This was a prerequisite for the traditional communities before the establishment of the state. The 1953 *Law on the Jurisdiction of the Rabbinical Courts* finally stipulated that marriage and divorce of Jews in Israel would come under the jurisdiction of rabbinical courts. They base their jurisdiction solely on the traditional interpretation of Jewish law, the Halakhah.^6^ Ever since its establishment, it was Haredi and religious Zionist men serving as judges, dayanim, of the state’s rabbinical courts.^7^ So, in matters of marriage and divorce, and conversion, Jews in the state of Israel are subject to the cultural norms of orthodox Judaism, which is constantly contested by Haredi Judaism to become even stricter. Thus, the marriage bans are applied in the state rabbinical courts to the entire Jewish population. In order to prevent “illegitimate” marriages, the Israeli State Chief Rabbinate regularly compiles and updates a blacklist with the names of persons who are not allowed to marry under Jewish law. Despite strong criticism from humanist organizations, this practice continues to this day. In spring 2017, there were 6787 people on the list covered by the Jewish marriage ban (Sharon 2017). Due to this situation, the population of Israel, including the Jewish, are denied the human right to marry “without any limitation due to race, nationality or religion” (Article 16, Declaration of the Human Rights) and to found a family.

The lack of civil marriage in Israel and the hegemony of the Haredim and the religious Zionists in the state rabbinical courts, are causing a growing number of non-Jewish and Jewish Israeli citizens to marry abroad (CBS 2015).^8^ Based on a precedent, since 1963 the Ministry of Interior officially recognizes marriages of its citizens abroad.^9^ After such a marriage, non-Jews can obtain Israeli citizenship, though the “national status” of the newly wed does not change and non-Jews still have to undergo conversion in order to become citizens with full legal rights. Divorces form another problem for Jewish citizens in Israel. According to the orthodox regulations, a divorce can be only proceeded, when both sides agree to hand out the divorce document, the Get. While a wife’s refusal can be overruled by the rabbinical judges, a husband’s refusal to give the Get, can lead to long years of legal bondage of the woman. There are numerous cases every year in Israel of such unsolved divorce procedures (Dödtmann 2021, p. 195).

Attempts to establish civil marriage in Israel have all been thwarted by the civil Supreme Court, which has confirmed the jurisdiction of the rabbinical courts for marriage and divorce according to the Status quo. The Haredi authorities and their representatives strictly reject the possibility of civil marriage in Israel. This, they argue, would lead to an increase in mixed marriages and thus to the assimilation of Israeli Jews with the non-Jewish environment, which must be prevented at all costs. Though a majority of secular Jews in Israel support civil marriage, the disapproval of intermarriage is also high among non-Haredi Jews.^10^

Jewish ultra-Orthodoxy is also actively intermingling with international Jewish identity politics. The orthodox hegemony in Israel hinders progressive Jewish denominations from freely developing. This is central for the religious confirmation of a person’s Jewishness, e.g. through conversion. The Supreme Rabbinical Court refuses to recognize people who have been trained in the Conservative rite (Masorti) or Reform rites and who have converted at a non-governmental institution. It even maintains a black list with the names of approximately 160 orthodox rabbis abroad who have been denied the legitimacy to testify to the Jewishness of a person (Maltz 2018). The conversion monopoly of the strict religious is the subject of ongoing legal and political disputes. So was the case in spring 2022, after the Russian invasion of Ukraine. Ukrainian Jews, trying to immigrate to Israel under the *Law of Return*, were put in legal suspense because of not having converted under the auspices of rabbis who got the approval by Israeli Haredi socialized state rabbis (Maltz 2022).

## The private religious courts of the Haredim

Based on the *Law for Arbitration* from 1968 there exists another legal framework in Israel which runs contradictory to civil decision making—the private religious court system. Each Haredi sub-stream runs its own Beit Din LeTzeddek, in short Badatz, which deals with purity (Kashrut), property (Mamonim) or civil issues. In their choice of legal means, Haredi Jews find themselves in a normative and cultural conflict. According to a survey from 2009, over 85% of the Haredim considered the Jewish principle more important than the democratic one in a dispute (Arian and Kisser-Shemgar 2009). Thus, they feel more committed to the traditional religious court system than to the state system, which follows the “Law of the Gentiles.” However, in heavily contested issues, many ultraorthodox Jews and even the Haredi rabbinical leadership prefer independent state courts to solve a case, for the religious courts are all related to a certain Haredi subgroup.

Still, for Haredi Jews a Badatz is the first address for legal battle. It derives its legitimacy from the obedience required in Haredi society to hear disputes only within their own religious system and not to hand over any Jew to a “Court of the Gentiles.” All legal matters, should be resolved within the Jewish community, according to the *Shulchan Aruch*, one of the most authoritative collections of laws of the late Middle Ages. To approach a non-halakhic judicial system without the consent of a rabbi or to report offenses committed by a Jew—as in cases of domestic violence, tax evasion, or corruption—is considered illegitimate denunciation. A whistle-blower is called a “Mossér,” an informer, who by his actions puts other Jews in danger. The halakhic judgment on a “Mossér” is social ostracism. In matters of averting great harm to the Jewish community, the “Din Roddef,” the “judgment on the persecutor,” can be applied, i.e., the denunciator should be banned or even killed.

Before a Haredi Jew goes to a state court, he must obtain the consent of a rabbi or rabbinical court, otherwise this step is considered a serious offense against the norms of Haredi society and the person concerned may be branded by the community as an “Arur,” a “cursed one.” Only in the case of capital crime like murder, the direct involvement of state authorities is permitted. The claim to sole representation, also for civil law matters, means that defendants who do not wish to appear before a rabbinical court in a dispute are given the halakhic regulatory punishment of the banishment letter for doing so, the “Ktav Serúv.” The person so punished is then ostracized from the Haredi community until he or she agrees to stand trial before the rabbinical court. Although the state Supreme Court ruled as early as 1996 that this means is illegal, the banishment letter continues to be used within the Haredi community in the 21st century.

## Haredi jewry and the Shabbat in Israel

For Haredi Jews, observing Shabbat is the essential religious obligations, based on the biblical commandment “Remember the Shabbat to keep it holy” (Exodus 20: 8–11). Failure to observe the holy day, a Shabbat desecration, is considered a capital crime and punished with exclusion, ostracism, or denunciation. The state of Israel has committed itself to respecting Shabbat as a Jewish day of rest and to enforce it by law (Shetreet and Homolka 2017). The 1951 *Law on Hours of Work and Rest Periods* regulates the Shabbat as a weekly holiday in Israel. In it, the Knesset stipulates that a Jew is entitled to at least 36 h of continuous rest from work and prohibits Jews from working. However, the Ministry of Economy and Industry may grant exemptions, e.g. at the end of 2015, there were a total of 342 such exemptions in the areas of security, work processes, and services vital to the public. A second legal way for Jews to perform work on Shabbat is opened up by the *Decree to Municipalities*. This law delegates oversight of the opening and closing of stores, restaurants, cafes, cinemas, theatres, and other establishments on Shabbat to local authorities. As a result, many secularly dominated municipalities created their own legally controversial bylaws, under which restaurants, zoos, cultural and sporting events may operate as permissible amusements on Shabbat in their administrative areas. Religiously dominated communities, in turn, waive the freedoms granted in the directive. A third law affects work and public life in Israel on Shabbat: the 1961 *Directives on Transportation*, which stipulated that public buses and trains may not operate on Shabbat and Jewish holydays—with the possibility for the Ministry of Transportation to give special allowances (Finkelstein 2016).

On closer examination, the legal provisions governing Shabbat in Israel are a *fictio iuris. *It is a permanent source of conflict between the state, the secular population and the Haredi community. On the one hand, the ban on Jews opening stores and working on Shabbat is one of the strictest legal regulations in the world. It is intended to emphasize the “Jewish character” of the state of Israel. On the other hand, the numerous exceptions, especially in the *Law on Hours of Work and Rest Periods*, mean that the legislation is constantly being circumvented. The exceptions take into account a reality in which public life in Israel is maintained to some extent even on Shabbat and other religious holidays, and the cultural norms of the Haredim are not applied.

Consistently over ten percent of Jewish workers also work on Shabbat (Eliash et al. 2014). For Haredi society, this percentage of working Jews represents a continuous Shabbat desecration. The conflict over its observance has been smouldering in Israel for decades. The genesis of this inner-Jewish conflict is very present on both sides. The first, sometimes violent clashes between secular and strictly religious Jews between the 1920s and 1950s went down in the history books as the “Jerusalem Shabbat Wars” and ended with the cultural hegemony of the Haredi Jewry over Jerusalem’s ultraorthodox quarters around the legendary Kikar HaShabbat (Friedman and Shelhav 1985). Since then, an ever-growing number of city districts and communities with a Haredi majority apply to the strict Shabbat rules and close off entire neighbourhoods and towns.

Over the past 20 years, transport policy has become the central point of contention between the secular and the Haredi population. In Israel’s cities and metropolitan areas, the proportion of young, secular residents and commuters is growing. There is great pressure on local politicians to provide solutions for better public mobility, including on Shabbat. This provokes sharp opposition from the Haredi community, which due to lower employment rate has relatively few commuters and few car owners. When in February 2012, the Tel Aviv-Jaffo city council, a municipality with approximately 40% (mostly secular) car-less households, attempted to obtain a permit from the Ministry of Transportation for public bus routes operating on Jewish holidays, Haredi spokespeople pointed, that this would break a taboo and harm the Status quo. Israel Me’ir Lau, former state-appointed rabbi of the city, said, “We need an expression that the state is Jewish and that the city of Tel Aviv is Hebrew. Just like 50 years ago. Today there are so many cars. […] Why is it not possible today? Shabbat was a unifying moment—for the family and for the whole population. We should not be like any other state! […] To the Minister of Transport and to the Minister of the Interior: (We must) keep Shabbat, as befits a Jewish state” (Reshet Bet 2012). Even though there is minor progress on a local level, lack of public transportation on Shabbat and Jewish holidays, is still exceptional in an industrialized country. It is another expression of the hegemony and religious coercion based on the Status quo.

## Conclusion

Over the last decades, the state of Israel and the leadership of the Haredi communities have been creating a situation, where they seemingly indissoluble depend on each other. By the *Status quo* and by the definition as a Jewish nation-state, Israel has put itself in the position where it can ignore liberal principles of equal rights for its citizens and gives some legal means into the hands of a religious fundamentalist group to discriminate against women and minorities in particular (Barzilai 2004). Haredi (and religious Zionist) Judaism function as guardians of the borders of the Jewish collective. By its hegemony over the question “Who is a Jew?”, Jewish religion in its orthodox interpretation can function as a legalistic shield for the exclusivist politics of Israeli Jewish ethno-nationalism (Ram 2008, p. 62), for the state sees itself as property of the “Jewish people” resp. the “Jewish nation,” but not as that of its citizens.

Israel bestowed ultraorthodox Judaism with material, cultural and political sources, that it never possessed before. The Haredi community, whose continued existence after the Shoah had been doubted from many sides, has gone through an impressive phase of consolidation and strengthening in the course of the over 70-year existence of the state of Israel. With state support, Haredi Jewry was able to establish a powerful autonomous education system which enabled the development of a new Jewish “Society of Learning Men,” that puts all efforts into a “pure” religious, isolationist lifestyle. State support and legal preference of the cultural minority of the Haredim maintain and strengthen their status of cultural insularity.

Even though there exist strong frictions over the questions of military service, of integration into the workforce, and of Shabbat regulations, the state, i.e., its execution and jurisdiction, and Haredi society are eager to preserve the *Status quo *against the contest of liberal, democratic ideas, which demand the dismantling of the theocratic and discriminatory aspects of Israeli legislation. Therefore, the majority of the Haredim in Israel in early 21st century accepts the idea of a Jewish nation-state, although it continues to reject the concept of secular nationalism. In this, it behaves like other contemporary “religious rebels” of post-modernity (Juergensmeyer 2008, p. 6). They reject the notion of Western nations that a state can only be understood as a matter of secular contract. But they have common ground: For Haredi Jews, as for secular and religious Zionists, the ultimate legitimation for the claim to ownership of the “Land of Israel” remains the Holy Scriptures, the Torah. With the demographically rising importance of Haredi Judaism, it can be predicted, that this fundamentalist religious streaming will possibly obtain even more political power within the decades to come. Then, the question will be, whether the concerted forces of fundamentalist Judaism in Israel—Haredi Jewry and militant religious Zionism—will strive for the reshaping of a theocratic state and by that fulfill the vision of modern religious fundamentalism: restoring the sole rule of religion against secularism?

## Footnotes

1 The state of Israel does not have an official constitution, instead it conducts jurisdiction on the basis of so-called Basic Laws. The most prominent and most recent of a Basic Law defining Israel as *Jewish-nation state* is the *Basic Law: Israel as the Nation-State of the Jewish People*, 19 July 2018 (7 Av 5778).

2 For a discussion about the idea and the territory of the Land Israel see Sand, Shlomo. 2012. *The Invention of the Land Israel: From Holy Land to Homeland*. London and New York: Verso Books.

3 Israel’s Basic Laws: The Law of Return. 5 July 1950. In an amendment from 1970, the right to citizenship was extended to people with one Jewish (male or female) grandparent and to a person who is married to a Jew. Amendment No. 2, 5730–1970.

4 “Speech of the dassies and pigs,” 26 March 1990. According to Jewish religion, dassies and pigs are impure animals and Jews are forbidden to breed or eat them. Quoted after: Kimmerling (2004, p. 237).

5 Ibid.

6 Law of the Rabbinical Courts—Marriage and Divorce, 5713–1953.

7 In contrast to the state Sharia courts, which rule over personal matters of Israeli Muslims, where in spring 2017 a female judge was nominated for the first time.

8 In the year 2013, 17% of marriages of Israelis were proceeded abroad. Central Bureau of Statistics. (2015). *Collection of Data on Occasion of Tu beAv—2013*. 29 July 2015. http://cbs.gov.il/reader/newhodaot/hodaa_template.html?hodaa=201511200 (Accessed 21 December 2021).

9 HCJ 143/62. Funk Schlesinger v. Minister of Interior. 22 February 1963.

10 Surveys show that up to two-thirds of Jewish Israelis interviewed considered “assimilation an existential threat to the Jewish people.” Cf. Haaretz. *Poll: Most Israelis Oppose Intermarriage*. 22 August 2014. https://www.haaretz.com/israelnews/1.611928 (Accessed 21 December 2021); The Times of Israel. *Most Israelis support marriage freedom, civil marriage*. 14 February 2017. https://www.timesofisrael.com/most-israelis-support-marriage-freedom-civil-marriage-poll/ (Accessed 21 December 2021).
